# The efficacy and safety of neoadjuvant nimotuzumab for gastric cancer: A meta-analysis of randomized controlled studies: Erratum

**DOI:** 10.1097/MD.0000000000028670

**Published:** 2022-01-21

In the article, “The efficacy and safety of neoadjuvant nimotuzumab for gastric cancer: A meta-analysis of randomized controlled studies”,^[1]^ which appears in Volume 100, Issue 50 of *Medicine*, the authors Bin Cao and Qi Zhang have been added. The corresponding author has been updated from Hui-qing Zhang to Qi Zhang.
